# Rural Clinician Scarcity and Job Preferences of Doctors and Nurses in India: A Discrete Choice Experiment

**DOI:** 10.1371/journal.pone.0082984

**Published:** 2013-12-20

**Authors:** Krishna D. Rao, Mandy Ryan, Zubin Shroff, Marko Vujicic, Sudha Ramani, Peter Berman

**Affiliations:** 1 Public Health Foundation of India, New Delhi, India; 2 Health Economics Research Unit, Institute of Applied Health Sciences, University of Aberdeen, Aberdeen, Scotland; 3 Department of Global Health and Population, Harvard School of Public Health, Boston, Massachusetts, United States of America; 4 American Dental Association, Chicago, Illinois, United States of America; 5 Indian Institute of Public Health (Hyderabad), Hyderabad, India; 6 Department of Global Health and Population, Harvard School of Public Health, Boston, Massachusetts, United States of America; University of Alabama at Birmingham, United States of America

## Abstract

The scarcity of rural doctors has undermined the ability of health systems in low and middle-income countries like India to provide quality services to rural populations. This study examines job preferences of doctors and nurses to inform what works in terms of rural recruitment strategies. Job acceptance of different strategies was compared to identify policy options for increasing the availability of clinical providers in rural areas. In 2010 a Discrete Choice Experiment was conducted in India. The study sample included final year medical and nursing students, and in-service doctors and nurses serving at Primary Health Centers. Eight job attributes were identified and a D-efficient fractional factorial design was used to construct pairs of job choices. Respondent acceptance of job choices was analyzed using multi-level logistic regression. Location mattered; jobs in areas offering urban amenities had a high likelihood of being accepted. Higher salary had small effect on doctor, but large effect on nurse, acceptance of rural jobs. At five times current salary levels, 13% (31%) of medical students (doctors) were willing to accept rural jobs. At half this level, 61% (52%) of nursing students (nurses) accepted a rural job. The strategy of reserving seats for specialist training in exchange for rural service had a large effect on job acceptance among doctors, nurses and nursing students. For doctors and nurses, properly staffed and equipped health facilities, and housing had small effects on job acceptance. Rural upbringing was not associated with rural job acceptance. Incentivizing doctors for rural service is expensive. A broader strategy of substantial salary increases with improved living, working environment, and education incentives is necessary. For both doctors and nurses, the usual strategies of moderate salary increases, good facility infrastructure, and housing will not be effective. Non-physician clinicians like nurse-practitioners offer an affordable alternative for delivering rural health care.

## Introduction

Health systems in low and middle-income countries (LMIC) like India struggle to provide quality clinical services to rural populations. An important constraint facing these countries is the scarcity of physicians in rural areas. In India, for example, almost 60% of health workers reside in urban areas even though 74% of the country’s population is rural [1]. This rural scarcity is particularly stark when compared to the urban availability of clinical care providers. In rural (urban) India there are 1.2(11.3) physicians and 0.7(4.3) nurses and midwifes per 10,000 population [1].

Efforts to reduce the scarcity of rural clinicians in LMICs have broadly focused on three strategies. One is to make rural service compulsory; however, this has generally been unpopular and unsuccessful [2]. A second strategy is to make rural service more attractive by offering monetary and non-monetary incentives. Several studies have shown that job choices are driven by salary, as well as, non-monetary job characteristics such as living conditions, educational opportunities for children, opportunities for training, and future career prospects [3]–[7]. A third strategy of task shifting involves deploying non-physician clinicians (e.g. nurse-practitioner or medial assistant) to perform many of the functions of a doctor [8]. Studies have shown that non-physician clinicians can perform comparably with doctors in primary care settings [9]–[13].

Indian strategies to improve rural recruitment have been born out of an administrative response, rather than, from a systematic understanding of health worker preferences [14]. This has prevented policy makers from taking a comprehensive view of the rural recruitment problem. Moreover, strategies that have been attempted like higher salaries for rural postings, admission to specialist training after some years in rural service, and housing have been offered as singleton incentives (typically only salary increases) [15]–[16]. While task shifting for primary care has been attempted on a limited scale, concerns about quality of care have contributed to restricting further expansion [17]. In general, little evidence exists on the effectiveness of individual rural retention strategies or how they can be improved. Importantly, current strategies mostly focus on doctors with little information on nurses. Such approaches based on a narrow view of the rural recruitment problem have limited the range of strategies that have been attempted.

This study aims to better understand ‘what works’ in terms of strategies for increasing the presence of qualified clinical care providers in rural areas of India. This is approached from the perspective of job preferences of both trainee and in-service doctors and nurses. This study has the following specific aims. First, to identify effective rural recruitment strategies based on health worker job preferences. Second, to compare how the effectiveness of these strategies differs between doctors and nurses. Third, to outline policy options for increasing the availability of clinical care providers in rural areas. The study focused on final year medical and nursing students, as well as, doctors and nurses serving at Primary Health Centers (PHC) and sub-district hospitals. The focus on final year students is because they will soon enter the job market, and on doctors and nurses is because they both provide clinical care at PHCs. The two Indian states where this study was conducted represent diversity both in terms of geography and in their capacity to produce doctors and nurses.

To elicit health worker preferences for different job attributes we use the Discrete Choice Experiment (DCE) methodology. The DCE method has been used extensively in the health economics field [3]–[7], [18]–[21]. To the best of our knowledge, this is the first DCE to be conducted in India for this purpose. Importantly, this study is one of the few that compares the stated job preferences of doctors and nurses for rural recruitment in the same setting.

## Methods

Ethical approval was obtained from the Ethical Review Committee of the Public Health Foundation of India.

The Discrete Choice Experiment (DCE) method is a quantitative technique that aims to elicit stated preferences of individuals [19]. This technique helps to uncover how individuals’ value particular attributes of a job by asking them to state their preferred choice over hypothetical job alternatives.

The DCE methodology has its theoretical foundations in the random utility framework [21]. A respondent (i.e. health worker) is assumed to choose among several alternative jobs. He or she will choose the job that produces the highest utility. The random utility framework assumes that the utility of a given job has two components - deterministic and random [21]. The deterministic component is a function of observable job attributes (e.g. pay, working conditions, location), each of which has a ‘ utility weight’ (see equation 1). The random component is determined by unobserved job attributes in addition to individual-level preference variation. The utility derived from a job is not directly observed, implying that the utility weights of the job attributes cannot be directly estimated. In the DCE methodology, when respondents choose their preferred job from a set of alternative jobs, the probability of choosing a job can be estimated (see equation 2). And after making certain assumptions, the job attribute utility weights can be estimated using standard econometric techniques [21]. It should be noted that an underlying assumption of these models is that individuals have a complete ranking of job opportunities that is determined by their preferences for the varying job attributes.

### Questionnaire Development and Administration

A qualitative study was conducted between January and July 2010 in the two study states to identify what job attributes were important for trainee and in-service doctors and nurses [22]. A total of 80 in-depth interviews were conducted with medical students, nursing students, and doctors and nurses in primary health centers. A diverse set of job attributes was elicited and clustered based on the frequency with which attributes were cited in health worker interviews and from policy maker ratings on the ‘actionablility’ of the attribute. Table 1 describes the final set of eight job attributes and their levels.

**Table 1:** Attributes and levels [Reference category for each attribute highlighted in italics].

	Attribute	Levels
1	Type of health center	*1. Clinic*
		2. Small hospital (20–30 beds)
		3. Large hospital (50–100 beds)
2	Area	*1. Located in a poorly connected place with bad education facility for children and poor housing provided*
		2. Located in a poorly connected place with bad education facility for children but good housing provided
		3. Located in a well-connected place, having good education facilities for children but poor quality housing provided
		4. Located in a well-connected place, having good education facilities for children and good quality housing provided
3	Health center infrastructure	*1. Building in poor condition, inadequate equipment, and frequent shortages of supplies and drugs*
		2. Well maintained building, adequately equipped with few shortages of supplies and drugs.
4	Staff	*1. Few staff and heavy workload*
		2. Fully staffed and moderate workload
5	Salary (including allowances, Rs/month)	1. Doctors: *30,000* 45,000 65,000 80,000
		2. Nurses: *10,000* 15,000 25,000 30,000
6	Change in location to city/town	*1. Uncertain*
		2. On completion of 3 years
7	Professional development	*1. Short duration training courses for skill development*
		2. Easier admission to PG after 3 years of service in same job through reservation/quota.
8	Job location	*1. The job is not located in your native area*
		2. The job is located in your native area

Source: Study data.

Given the number of attributes and their associated levels, a total of 1,536 (2^5^*3^1^*4^2^) unique jobs can be derived from different combinations of these attributes and levels. To limit the number of job choice sets to 16 (which is generally the convention for DCE studies), a statistically efficient (D-efficient) fractional factorial design was used. This ensures minimum variation around the parameter estimates by minimizing the estimated standard errors. SAS software was used to generate the design [23].

Respondents were presented with a set of 16 choice sets, each choice set containing a pair of jobs that had the same attributes but not all at the same level. The survey used a two-stage response design in which the respondent first made a choice between the presented job pairs by responding to the question “*Which of the two jobs do you prefer*”. In the second stage, medical and nursing students were first asked “*Will you choose this job if it was offered to you*”, and in-service doctors and nurses were asked “*Will you choose this job over your current job*”. The second stage serves as a ‘opt-out’ option and offers respondents the opportunity to reject the forced choice made in the first stage. Forcing choice to be confined to the first stage can potentially bias respondent preferences for job attributes [21].

Four additional choice sets were inserted. Two of these were dominance tests, in which one job was superior to the other in terms of all attributes, and expected to be chosen by a rational respondent. For the attributes “place of work” and “location of job”, no level was consistently considered to be dominant over the other, and hence, for the rationality test, the level of these two attributes was kept same in job 1 and job 2. Another two choice sets were added as ‘hold outs’, responses to which enabled evaluation of the model’s predictive accuracy.

The questionnaire was administered in English to medical students and doctors and in the local language to nurses and nursing students.

### Sample Selection

The target sample (size) was final year undergraduate medical students (150), final year GNM nursing students (150), in-service doctors (150) and nurses (150) working at PHCs.

#### Medical and nursing schools and students

The student sample was from the state of Andhra Pradesh. The selection of medical and nursing students was a two-step process- first; medical/nursing schools were purposively selected, followed by a convenience sampling of final year medical and nursing students. One medical and nursing school was selected from each of the three regions of the state in such a manner that the aggregate sample of colleges had representation from public and private colleges, urban and rural locations and a range of academic reputations. A total of 4 (3 public and 1 private) medical and 4 nursing (2 public and 2 private) schools were selected. Within each school, students in their final year – fourth year MBBS students and second year General Nursing and Midwifery (GNM) students- were invited to participate in the study by the school administration. The majority of students in the class accepted and participated. No information on non-participants was collected. Among medical students willing to participate, an equal number of male and female students were administered the questionnaire. Informed written consent was obtained from all respondents.

#### In-service doctors and nurses

To select in-service doctors and nurses employed at PHCs in Andhra Pradesh, one district from each of the state’s three regions was randomly selected. All candidates from the selected districts who were working in PHCs were invited to participate in the study. The majority of nurses and doctors in the district accepted and participated. No information on non-participants was collected. In Uttarkhand, a listing was made of the number of sanctioned posts for doctors and nurses at PHCs in each district of the state. Nurses posted at sub-district hospitals were also included in the sample. Six districts with the largest number of sanctioned posts for Medical Officers were selected from the two regions of the state. All eligible candidates from the selected districts were invited to participate in the study. Informed written consent was obtained from all respondents.

### Statistical Analysis

Data collected from the field survey was cleaned and double entered into a CSPro version 4.1 (US Census Bureau) database. All analysis was done in Stata 12 [24]. All analysis was stratified by students, in-service doctors, and nurses.

Analysis of the information collected included presenting summary statistics of respondent characteristics. Individuals who “failed” both dominance tests were dropped from the analysis.

In the DCE, each respondent stated their choice between 16 pairs of job choices. Further, respondents were also selected from a common school or district. Therefore the study data was hierarchically structured with the possibility of data being correlated within each level of hierarchy – there could be correlation in the job choice responses belonging to the same individual (intra-respondent), as well as, correlation among responses of individuals in the same school or district (intra-location). The outcome of interest is if the respondent accepted the job that he/she selected in the first stage. The binary response to the following questions were modeled: for medical and nursing students -“*Will you choose this job if it was offered to you*”, and in-service doctors and nurses - “*Will you choose this job over your current job*”. Multi-level logistic regression was used to model the binary responses related to job acceptance [25]–[27]. Models were run separately for medical students, nursing students, doctors and nurses. Because students were sampled within schools and in-service respondents from the same district, we also ran three-level models in which school/district was the third level to accommodate the intra-school or intra-district correlation. If these correlations were found to be significant then school or district level random effects were also included in the model.

The utility or satisfaction derived from job defined by choice-set i and by person j is represented by a continuous variable, 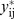 [28]. In the two level random effects linear regression framework shown below, where choice-sets are at level 1 and individuals at level 2, we can model 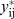 as:
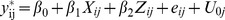
(1)where, 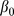 is the constant term, 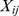 is a vector of job attribute-levels (see Table 1) and 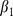 is the vector of regression coefficients associated with these attribute levels. 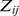 is a vector of individual-level characteristics (see Table 2), and 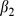 is the vector of regression coefficients associated with these characteristics. 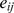 is the choice-set level error term, 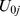 is the individual level random effects term and is assumed to be normally distributed with a mean of zero and a between-respondent level variance of 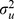.

**Table 2:** Sample description.

	Students		In-service	
	Medical	Nursing	Doctors	Nurses
Age (years)	21.90	20.38	35.98	32.43
	(1.20)	(1.62)	(7.41)	(7.38)
Male (%)	50	5	74	3
Rural upbringing (%)	12	73	28	51
Private school (%)	39	45	26	29
Years of service	N/a	N/a	5.78	6.61
			(4.55)	(6.60)
Sample size	161	132	221	232

Note: Figures in parentheses are standard deviations. N/a = not applicable.

Source: Study data.

We however only observe the binary variable, 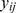, such that 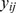 = 1 if 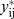 >0, and 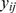 = 0 if 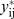
≤0. In the DCE context, 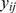 = 1 if person j accepts the job he/she selected in choice-set i. Otherwise, 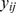 = 0. Conditional on 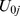 and assuming that 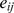 follows a standard logistic distribution [28], we model 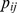 the probability that 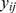 = 1, as follows:
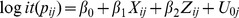
(2)where, 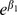 is the odds ratio associated with each attribute-level of the job. This odds ratio can be interpreted as how much more likely (if >1) or less likely (if <1) a job will be accepted when that attribute-level is present, compared to when it is not present, with other attribute-levels and covariates being at their reference categories. The vector of regression coefficients, 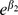 is the odds ratio associated with each individual characteristic. These odds ratios are interpreted as how much more or less likely a job will be accepted if that individual characteristic was present, compared to when it was not present, with other attribute-levels and covariates being at their reference categories.

The results from the logistic regression can be used to estimate the probability (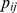) of accepting a job:
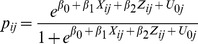
(3)


Equation (3) can be used to estimate the percentage of respondents who would take up a job for different combinations of job attribute-levels and individual characteristics. Wage supply elasticities were calculated by estimating the percentage change in the proportion of respondents willing to accept a job relative to the percentage change in salary.

The model’s predictive accuracy was measured by the area under the Receiver Operating Characteristic (ROC) curve, which gives an estimate of the percent of outcomes correctly predicted by the model [29]. The in-sample prediction is based on the range of data used to populate the model. The out-of-sample prediction was made with the help of the two holdout choice sets, which were not used in estimating the regression equations.

## Results

Of the 308 medical and nursing students who answered the questionnaire, 15 respondents who failed both the dominance (i.e. did not choose the job with better attribute levels) tests were dropped, reducing the sample size to 293 (161 medical and 132 nursing) students. The in-service questionnaire was administered to 457 doctors and nurses. After retaining those observations in the sample that met the sample eligibility requirements, and dropping four respondents that failed both the dominance tests the final sample size was 221 doctors and 232 nurses.

About half the medical students who participated were male with a mean age of about 22 years (Table 2). Nursing students were overwhelmingly female and were slightly younger. Only 12 percent of medical students had had grown up in a rural area compared to the majority of nursing students. Most of the medical school students and about half the nursing students were studying at government institutions.

The majority of doctors were male, had grown up in an urban area, attended public medical colleges, and had served for five years on average as Medical Officers. The overwhelming majority of nurses were female. Nurses tended to be younger than doctors, more than half of them had rural upbringing, and most of them had trained in government colleges. The mean duration of government service was similar for the sample of doctors and nurses.

The predictive accuracy of the model for medical students (80%), nursing students (72%), doctors (78%), and nurses (74%) indicated good model fit. The out-of-sample prediction using the two hold-out choice sets yielded the following proportions of responses correctly predicted– medical students (87%, 65%), nursing students (74%, 60%), doctors (90%, 56%), and nurses (77%, 70%). The statistical significance of the intra-cluster coefficient, rho, confirms the appropriateness of introducing the two or three-level random intercept model (see Table S1). Note that three-levels were included in the model only when the intra-school/district correlations were found to be significant.

**Figure 1:**
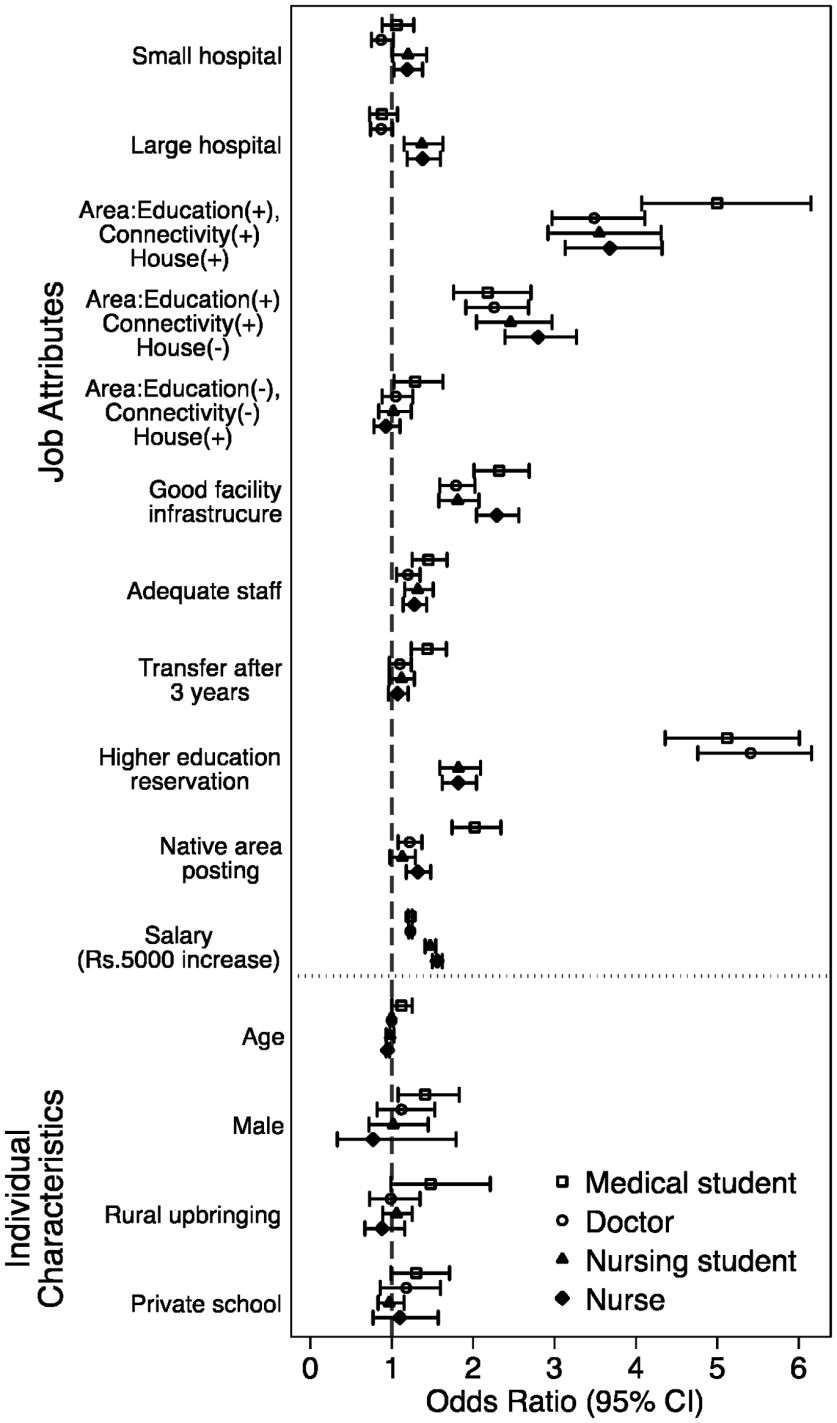
Determinants of job acceptance among trainee and in-service doctors and nurses.

Figure 1 presents the results from the multi-level logistic regression models. The plotted regression coefficients are in the form of odds-ratios and their 95% confidence intervals (Table S1). An attribute/characteristic is positively and significantly associated with accepting a job if the 95% confidence interval of the odds-ratio is greater than 1 i.e. above the vertical dashed line. The odds ratios represent the likelihood of accepting a job when that particular job attribute or individual characteristic is present in a reference case job. The reference case refers to a job in a clinic, located in an area with poor connectivity, poor education facilities for children, and poor housing; the clinic has poor infrastructure; it is poorly staffed and workload is high; transfer is uncertain; there is no reservation for in-service staff for higher education; job is not located in the respondent’s native area; and salary is entry level (Rs 30,000 for doctors and Rs 10,000 for nurses). In short, the reference case refers to a government job at a typical PHC located in a remote area with salary levels around what new recruits receive.

Among medical students and doctors, the likelihood of accepting a job, was significantly and positively (95% CI of odds-ratio> 1) associated with the following attributes - area (good education facilities for children and connectivity), good health facility infrastructure, adequate staff availability, guaranteed transfer after three years of service, reservation for higher education, posting in one’s native area, and salary. Job attributes that were not significantly associated were – a job in a small or big hospital, only having good housing in an area, and guaranteed transfer after three years (for doctors). For both medical students and in-service doctors a job with 20% higher salary was 1.23 (1.20–1.25) times more likely to be accepted, compared to a job with reference salary and other attribute levels. Notably, among all the job attributes, jobs with higher education training opportunities had the highest likelihood of being accepted. For instance, its effect size for medical students (OR = 5.12(4.36–6.01)) and doctors (OR = 5.41(4.76–6.16)) was greater than that of a job possessing the best area attribute. The likelihood of accepting a job diminished as the area became progressively ‘rural’. For example, medical students were five times more likely (OR = 5.00(4.07–6.05)) to accept a job in the ‘best’ area (i.e. good education facilities for children, connectivity, housing) or were twice as likely (OR = 2.18(1.76–2.17)) to accept a job in an area with only good education facilities and connectivity, compared to an area without these attributes. In general, none of the individual characteristics – being male (for medical students), rural upbringing, private medical school education – was significantly associated with the likelihood of accepting a job.

Among nursing students and nurses, the job attributes significantly associated with the likelihood of accepting a job included – job in a small or large hospital (as opposed to a health center), good area characteristics, good health facility infrastructure, adequate staff, reservation for higher education, posting in one’s native area (for nurses), and salary. Job attributes not significantly associated with job acceptance were- the availability of good housing only, guaranteed transfer after three years of service, and posting in one’s native area (for nursing students). Good facility infrastructure had the second biggest effect size after the best area attribute. For both nursing students and nurses a job with 50% higher salary was 1.47 (1.41–1.54) and 1.56(1.50–1.63) times more likely to be accepted, respectively, compared to a job with reference salary and other attribute levels. The likelihood of accepting a job diminishes as the area becomes progressively ‘rural’. None of the individual attributes (rural upbringing, private schooling, being male) were significantly associated with the likelihood of accepting a job. However, for nurses, being older significantly reduced the likelihood of accepting a job.

**Figure 2:**
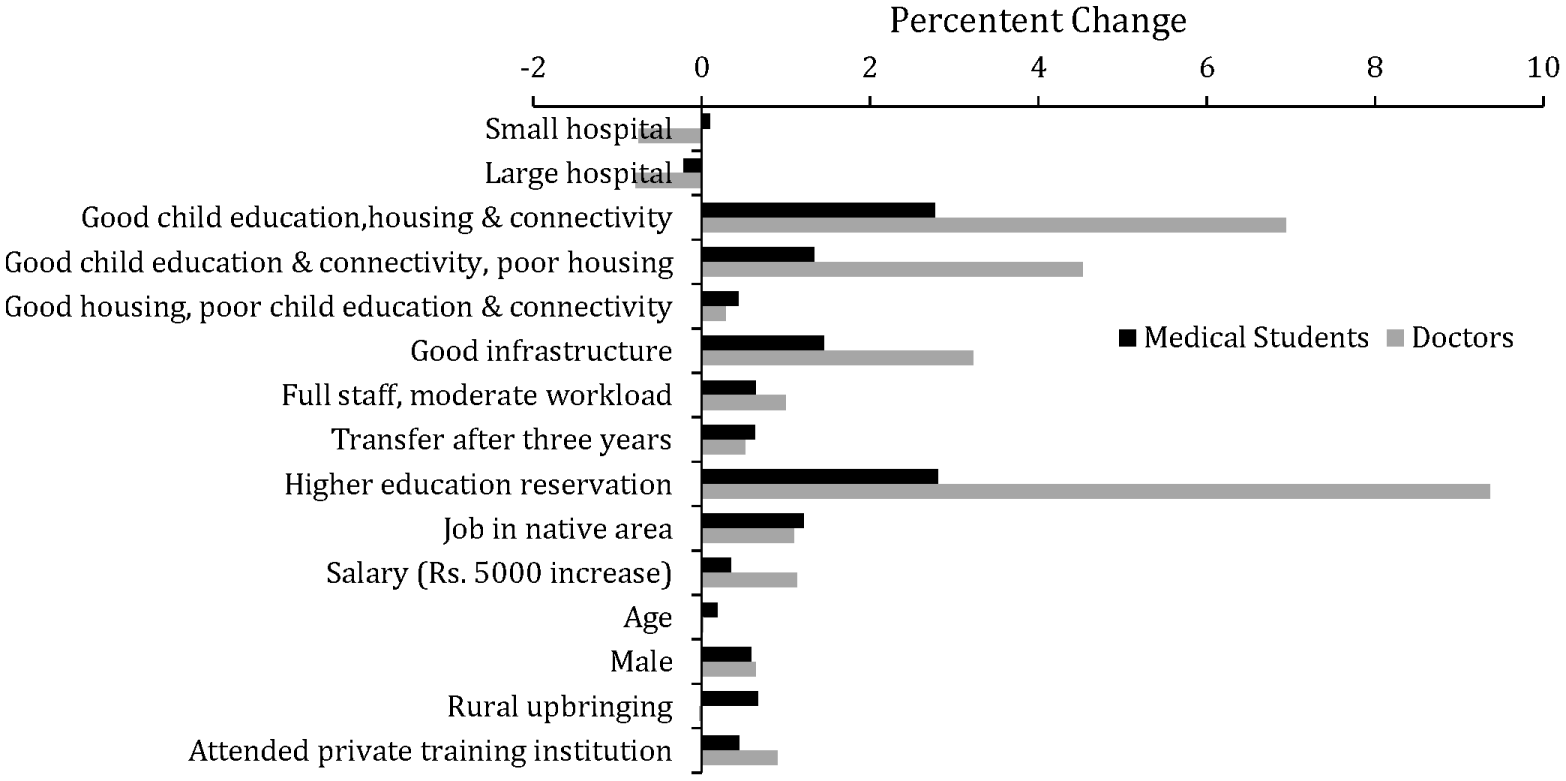
Percentage change (over base) in number of medical students and doctors willing to accept a rural job in the presence of specific job attributes and individual characteristics (base: salary Rs.30,000/month).

**Figure 3:**
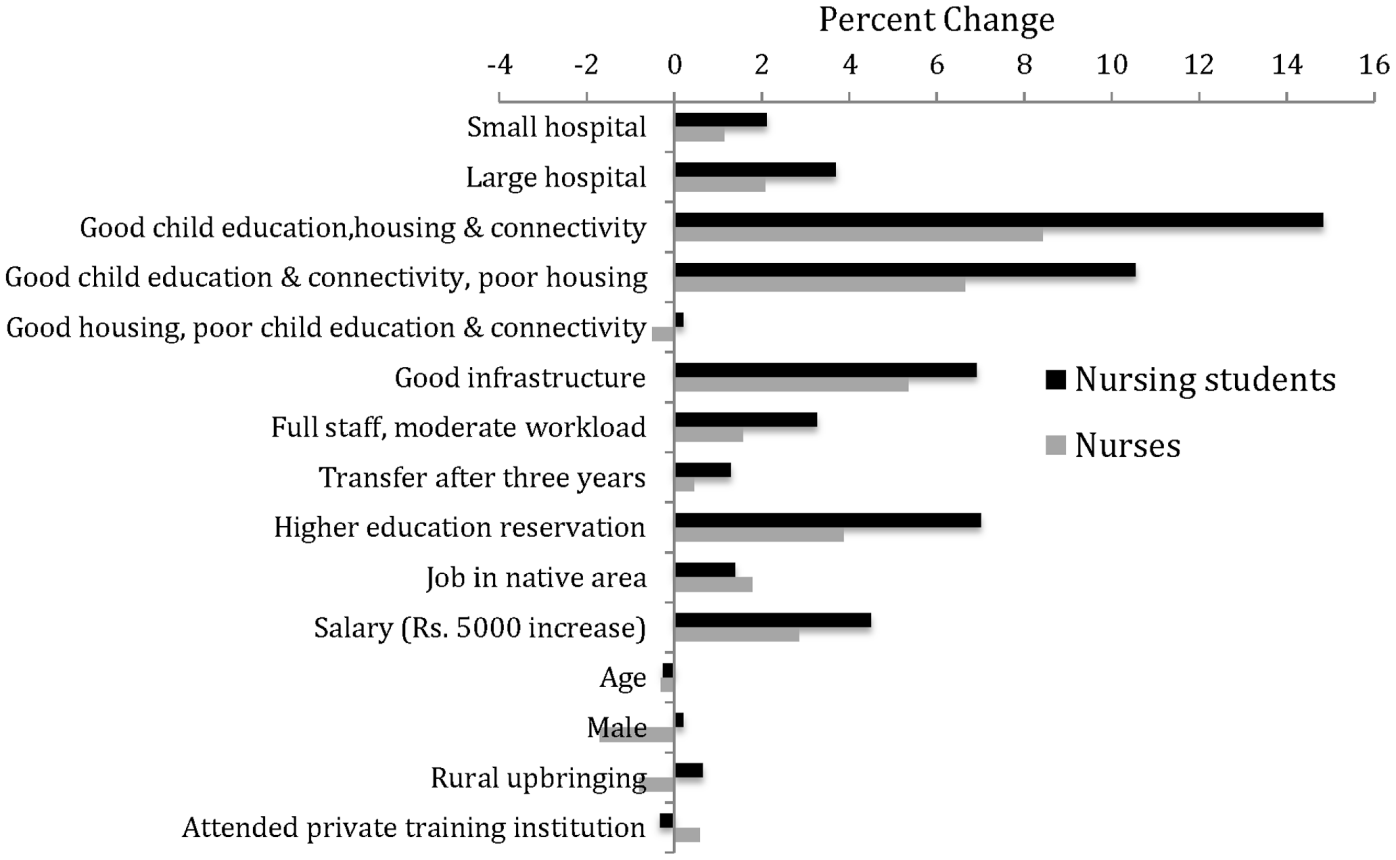
Percentage change (over base) in number of nursing students and nurses willing to accept a rural job in the presence of specific job attributes and individual characteristics (base: salary Rs.10,000/month).

Figure 2 (medical students and doctors) and Figure 3 (nursing students and nurses) shows the change in the percentage of respondents, over baseline (described earlier), who would accept a rural job in the presence of specific job attributes and individual characteristics (see Equation 3). Among medical students, the presence of different job attributes did not substantially increase the percentage of respondents opting for a rural job over baseline levels. For instance, in the presence of the best area attribute, 3% more medical students accepted a rural job, over baseline levels. For a job with reference case conditions and salary, less than 1.0% of medical students stated acceptance. In contrast, in-service doctors were more influenced by job attributes for taking up rural posts. For instance, 6.7% more doctors would take up a job if it possessed the best area attribute, and around 9.0% (2.8% for medical students) more would accept a rural job if offered specialist training in exchange for three years of rural service. Health facilities with good infrastructure would attract 3% more in-service doctors over baseline levels.

Job attributes had a larger effect on nursing students and nurse (Figure 3) uptake of rural jobs over baseline levels, compared to medical students and doctors. In the presence of the best area attribute, 15% (8%) more nursing students (nurses) would opt for rural service. For both nursing students and nurses, jobs in health facilities that had good infrastructure, and the presence of higher education opportunities (e.g. BSc nursing) has relatively large effects on increasing the percentage of respondents opting for rural jobs. For doctors and nurses, individual characteristics had small effects on increasing the percent of respondents opting for a rural job, over baseline levels.

**Figure 4:**
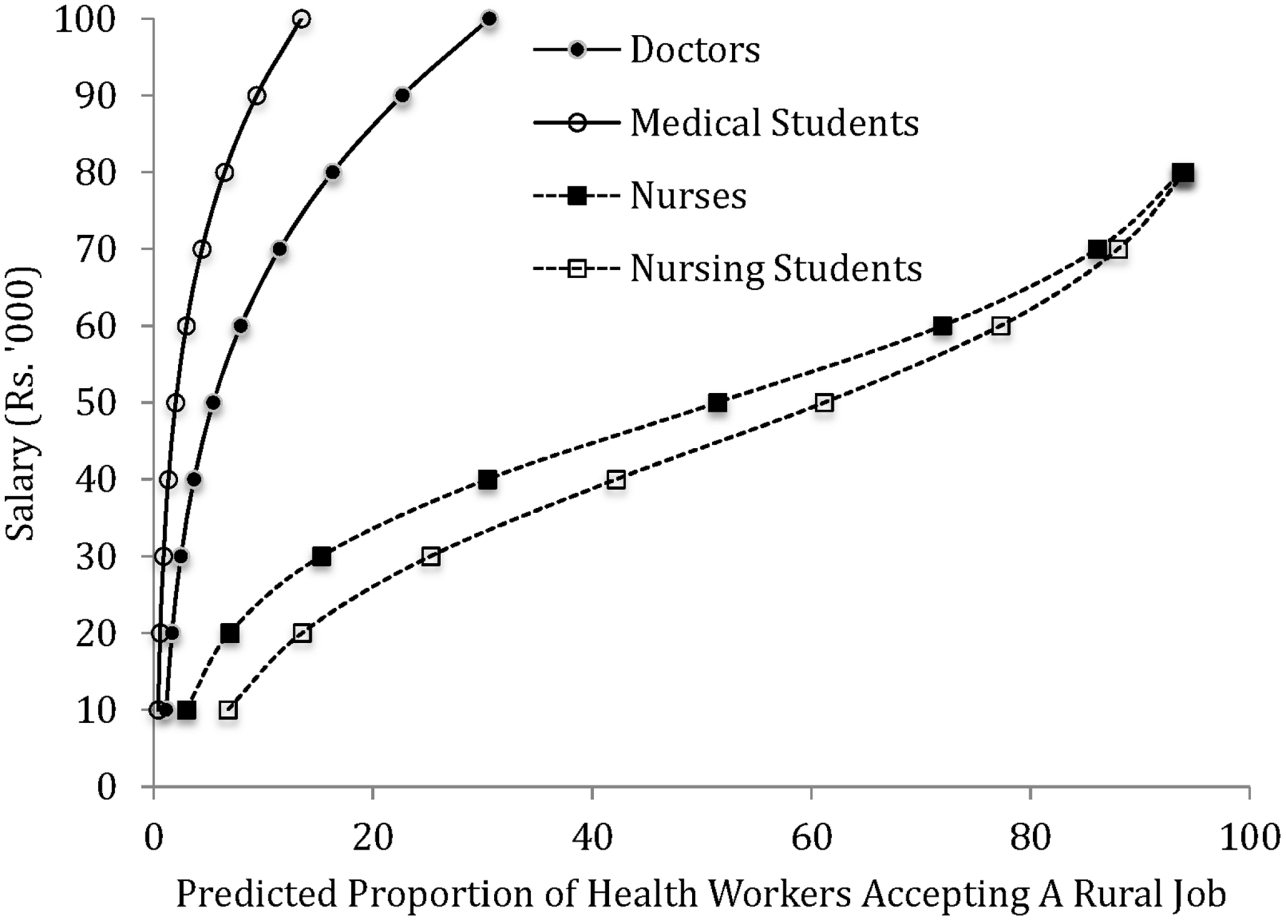
Supply of trainee and in-service doctors and nurses.

Figure 4 shows the labor supply curves for government jobs located in a typical rural area i.e. the reference case situation. Overall, for every salary level, a considerably higher proportion of nursing students and nurses were willing to accept a rural job compared to medical students and doctors. A doubling of salary from base levels increases the percentage of medical students (0.73% to 2%) and doctors (2% to 5%) available for rural service. In contrast, a doubling of salary has a greater effect on increases in the percentage of nursing students (7% to 14%) and nurses (3% to 7%) accepting rural jobs. At the high salary level of Rs. 100,000 per month, 13% (31%) of medical students (doctors) accepted a rural job. At half that salary level, 61% (52%) of nursing students (nurses) accepted a rural job.

Overall, the wage elasticity of supply of medical students and doctors was elastic over the salary rage. That is, the percentage increase in supply of health workers was greater than the percentage increase in salary. For nursing students and nurses, supply was elastic at lower salary levels and became inelastic towards the upper end of the salary range. Further, the supply of nursing students and nurses is more elastic, relative to medical students and doctors, at lower salary levels.

## Discussion

The DCE reported in this study highlighted several strategies available in LMICs like India for increasing the availability of clinical care providers in rural areas. Expectedly, location matters; for both trainee and in-service doctors and nurses, jobs located in an area with the ‘best’ attributes had one of the highest likelihoods of being accepted, and this declined as the area attributes became less desirable. This has several implications. For one, it highlights the importance of creating a good living environment for health workers in the area where they are posted. Since amenities like connectivity and children’s education are such important drivers of where health workers would like to work, effort should be made in providing such facilities where rural posts are located. The responsibility for creating such an environment will require an integrated approach that necessarily goes beyond the health ministry and include the education and rural development sectors in government. However, the cost of providing such an environment may ultimately be unaffordable to countries like India. Secondly, state health departments can expect greater success in posting doctors and nurses in rural posts that are close to urban settlements since the amenities of urban life are within reach. The greater concentration of doctors and nurses in semi-urban areas is testimony to this. However, for postings that are remote, a broader strategy of improving living environments is required. So, for instance, the common strategy of only providing higher salary or good housing will have limited success in making rural posts attractive.

In India’s context, reservation of specialist seats in exchange for a few years of rural service is a powerful strategy to attract and retain doctors (and nurses, and nursing students) to rural posts. Several states currently offer this scheme to their in-service doctors. In our study, this strategy had a large effect on the likelihood of selecting a rural job. For doctors, its effect on accepting a rural job was almost as powerful as a job in the ‘best’ area (i.e. having good connectivity, education facilities for children, and housing). This is expected since the pressing ambition of Indian medical graduates is to become a specialist, and the competition for specialist seats is intense due to the small number of seasts avaialble in comparison to the number of medical graduates. However, there is a larger question about how well qualified and motivated medical graduates are to serve as rural doctors.

For both trainee and in-service doctors and nurses, job attributes such as moderate salary increases, staffing health facilities adequately so that workloads are not overbearing, and good facility infrastructure, while important, did not have large effects on the likelihood of a rural job being accepted. In general, such singleton attributes are not very effective in increasing rural recruitment. However, when combined, they can have an additive effect- incentive ‘packages’ based on these attributes will be more effective in increasing rural recruitment than singleton incentives. This again reinforces the importance of taking a broader approach to designing strategies for increasing rural recruitment.

This study also highlights the relatively limited role that certain respondent characteristics play in uptake of rural jobs in India. Those who were brought up in rural areas were no more likely to accept rural jobs than those with an urban background. This suggests that a strategy of purposively recruiting students from Indian rural areas into medical and nursing schools may not be effective in increasing rural recruitment, and is somewhat at odds with previous research in other countries [13]. The findings do not provide any evidence that those educated at government medical colleges, where education is highly subsidized by the government, are more likely to accept a rural job than those educated at (more expensive) private medical colleges.

Incentivizing doctors to serve in rural areas is challenging and expensive. The supply of both student and in-service doctors for rural posts was not responsive to increases in salary, particularly at lower salary levels. The high salary levels at which a respectable proportion of medical students and doctors were willing to accept rural job is unaffordable for countries with limited health resources. In contrast, nursing students and nurses are more willing to accept a rural job, as well as, with greater enthusiasm in the presence of favorable job attributes. Moreover, the supply of nursing students and nurses is much more responsive to increases in salaries, particularly at lower levels, relative to medical students and doctors.

The nurses in this study were not trained to take on the clinical functions of doctors. However, their cadre offers the potential of becoming providers of basic clinical care. Several countries in Africa and Asia, as well as two states within India, have used nurse-practitioners or Medial Assistants to successfully staff primary care facilities [11], [16]. This potential of nurses or other non-physician clinician cadres, coupled with their greater propensity for rural service, makes them both a viable and affordable option for strengthening rural health services.

This study has several notable limitations. Because the DCE method relies on stated preferences, how health workers might behave (i.e. revealed preference) when actually faced with these incentives, can be different [22], [30]. Secondly, in the DCE health workers made a choice between hypothetical jobs. While every effort was made to make these job descriptions as realistic as possible in terms of their attributes and levels, nevertheless, they are limited in their realism. Third, respondents could be making job choices based on a few select attributes (e.g. salary) rather than assessing all the attributes. In this case preferences for select attribute-levels will be incorrectly interpreted as preference for all the attribute-levels that define that job. Fourth, one also needs to be cautious in interpreting the findings emerging from this DCE. Since the DCE focused on specific cadres (nurses and doctors) the findings are applicable to them. However, in a health system context where health workers operate in teams and not in isolation, it is usually difficult to change incentives (e.g. higher salary) for only some cadre without others also demanding improved compensation. This can rapidly become an expensive proposition for government. Moreover, certain strategies, such as reserving seats for specialist training, might not be feasible in certain contexts.

For countries like India that have limited health resources, creating a health system capable of providing reliable and quality clinical services in rural areas is an especially difficult challenge. Strategies that offer ‘packages of incentives’ that address both the professional and personal needs of doctors can improve their rural recruitment to a certain extent. Elements of this package should include, substantial salary increases, improvements to the living environment, provision of good children’s education, and where possible, reservation of seats for specialist training. However, pursuing this route to its full extent might ultimately be unaffordable and requires health departments to engage with other government departments (e.g. education, roads). The experience of several countries that have adopted non-physician clinicians as an alternative way of providing basic health services to rural populations offers important lessons for India’s own ambitions for achieving universal health coverage.

## Supporting Information

Table S1 **Multi-level Logistic Regression Results of Job Acceptance on Job and Individuals Characteristics.**
(DOCX)
