# Supplementary material for: Rural Clinician Scarcity and Job Preferences of Doctors and Nurses in India: A Discrete Choice Experiment
Source: PLoS One. 2013 Dec 20;8(12):e82984. doi: 10.1371/journal.pone.0082984 (PMC3869745; doi:10.1371/journal.pone.0082984)
Supplement: Table S1 — Multi-level Logistic Regression Results of Job Acceptance on Job and Individuals Characteristics. (DOCX) [file pone.0082984.s001.docx]

Table S1 Multil-level Logistic Regression Results of Job Acceptance On Job And Individuals Characteristics.


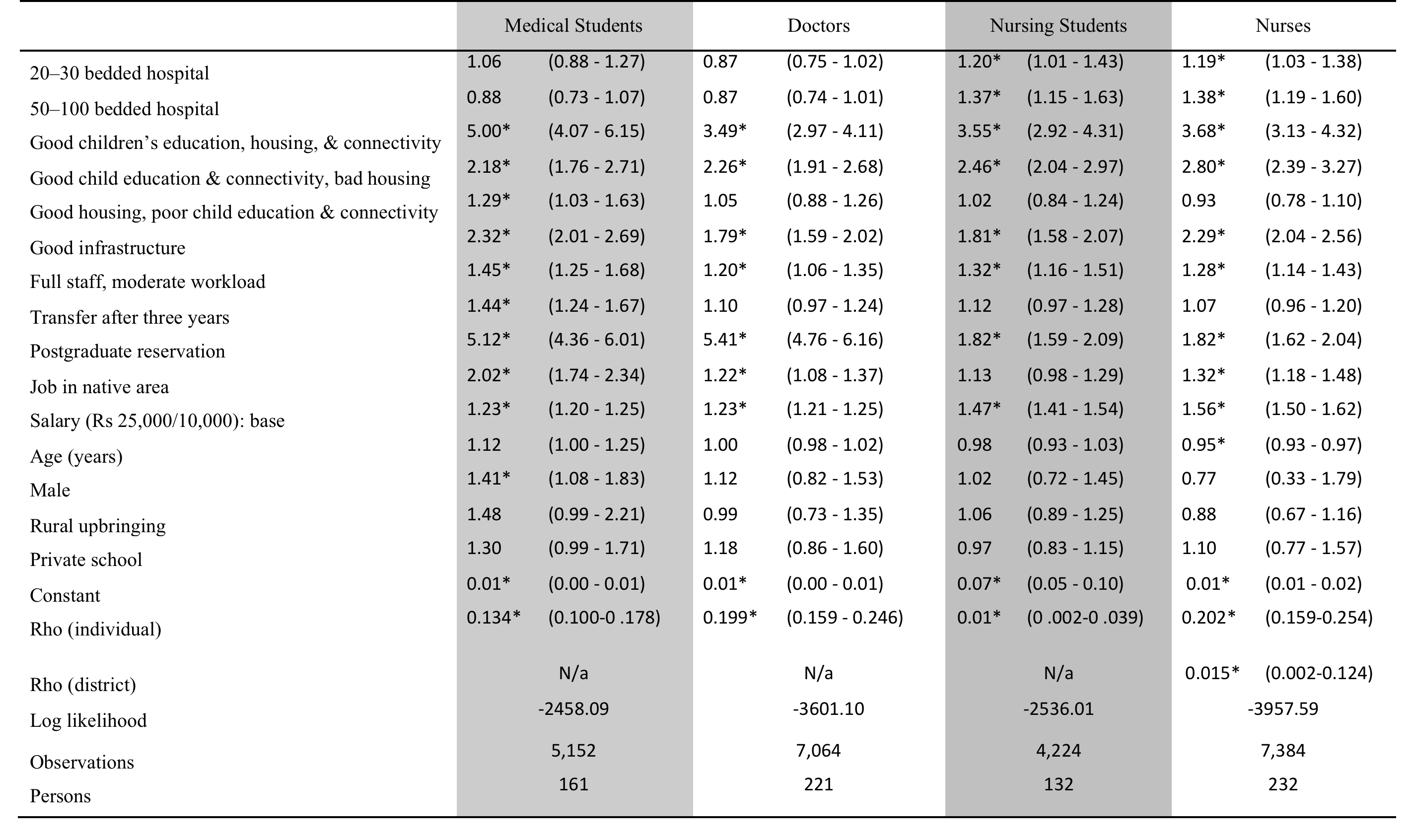


Note: (1) Figures are odds ratio (95% confidence intervals); (2) * Statistically significnat at 5% level.
